# Laser capture microdissection and cDNA array analysis of endometrium identify CCL16 and CCL21 as epithelial-derived inflammatory mediators associated with endometriosis

**DOI:** 10.1186/1477-7827-5-18

**Published:** 2007-05-17

**Authors:** Ashwini L Chand, Andrew S Murray, Rebecca L Jones, Natalie J Hannan, Lois A Salamonsen, Luk Rombauts

**Affiliations:** 1 Prince Henry's Institute of Medical Research, PO Box 5152, Clayton, Victoria 3168, Australia; 2 Department of Obstetrics and Gynaecology, Monash University, Clayton, Victoria 3168, Australia; 3 Division of Human Development, Academic Unit of Child Health, University of Manchester, St Mary's Hospital Research Floor, Hathersage Road, Manchester M13 OJH, UK; 4 Wellington School of Medicine, Otago University, Wellington, New Zealand

## Abstract

**Background:**

Understanding the pathophysiology of chemokine secretion in endometriosis may offer a novel area of therapeutic intervention. This study aimed to identify chemokines differentially expressed in epithelial glands in eutopic endometrium from normal women and those with endometriosis, and to establish the expression profiles of key chemokines in endometriotic lesions.

**Methods:**

Laser capture microdissection isolated epithelial glands from endometrial eutopic tissue from women with and without endometriosis in the mid-secretory phase of their menstrual cycles. Gene profiling of the excised glands used a human chemokine and receptor cDNA array. Selected chemokines were further examined using real-time PCR and immunohistochemistry.

**Results:**

22 chemokine/receptor genes were upregulated and two downregulated in pooled endometrial epithelium of women with endometriosis compared with controls. CCL16 and CCL21 mRNA was confirmed as elevated in some women with endometriosis compared to controls on individual samples. Immunoreactive CCL16 and CCL21 were predominantly confined to glands in eutopic and ectopic endometrium: leukocytes also stained. Immunoreactive CCL16 was overall higher in glands in ectopic vs. eutopic endometrium from the same woman (P < 0.05). Staining for CCL16 and CCL21 was highly correlated in individual tissues.

**Conclusion:**

This study provides novel candidate molecules and suggests a potential local role for CCL16 and CCL21 as mediators contributing to the inflammatory events associated with endometriosis.

## Background

Endometriosis is defined as the ectopic growth of endometrium-like tissue in locations outside the uterus including the ovaries, the uterine ligaments, the pelvic peritoneum, the Fallopian tubes and the external surface of the uterus. The mechanism/s by which endometriosis develops is still much debated; however retrograde menstruation and subsequent tissue persistence and proliferation is the most accepted theory. The complex question, why some women and not others, develop endometriotic lesions is likely to be due to differences in the local regulation of tissue proliferation, tissue remodelling and inflammatory processes. These observations also raise the question whether the current practice of prompt surgical or medical treatment is always appropriate.

Current evidence suggests that endometriosis-related pain and infertility result from local inflammation at the implant sites, with chemokine-induced recruitment and activation of immune cells [1]. Whilst some chemokines are produced constitutively, the majority (such as CXCL8 (IL-8) and CCL2 (MCP-1)) are induced upon cellular activation by inflammatory stimuli [2-9]. Constitutively expressed chemokines are believed to play a role in basal leukocyte trafficking and secondary lymphoid organ development while induced chemokines help marshal inflammatory, immune and angiogenic responses of the host [10-12]. It is now well established that chemokines also play a role in inflammatory pain sensation (nociception) and cell growth [13]. They are also pivotal in decreased immunologic surveillance, recognition and destruction of ectopic endometrial cells and perhaps facilitation of the implantation of ectopic endometrial tissues [14,15].

It is likely that the cascade of proinflammatory proteins secreted from endometriotic lesions and associated immune cells, dictate the extent of the inflammatory reaction linked with endometriosis, either by facilitating the survival of these lesions or by leading to their demise. Chemokines have been demonstrated to upregulate adhesion molecules and promote invasion of cancer cells [15-18]. Such actions could potentially facilitate the attachment and invasion of epithelial cells into the ectopic sites, as observed in cancer metastasis [19]. A number of chemokines have previously been implicated as mediators in the ontogenesis of endometriosis [20]. The levels of monocyte chemotactic protein-1 (MCP-1/CCL2) [7,8]; regulated on activation normal T-expressed and secreted (RANTES/CCL5) [21]; interleukin-8 (IL-8/CXCL8) [22,23]; and growth-regulated oncogene-α (GROα/CXCL1) [24] are elevated in the peritoneal fluid of women with endometriosis, and their concentrations correlate with the stage of the disease. Natural Killer (NK) cell activity is also mediated by chemokines and is decreased in patients with endometriosis [25].

Most studies have focused on the peritoneal fluid or serum concentrations of the chemokine of interest rather than its source. The latter may be important as the expression pattern of chemokines in the uterus is remarkably cell-type specific [26]. In addition, the cellular origin of chemokines also varies with cycle stage, although this also differs between individual cytokines [26,27]. For example, in the mid-secretory phase of the menstrual cycle, many cytokines are produced predominantly by the epithelial cells, while in the late secretory and menstrual phases, the same cytokines are also strongly expressed in both decidualised stromal cells and leukocytes [26,27], normal peritoneum, peritoneal fluid immune cells, and ectopic endometrial stromal cells [28]. This highlights the importance of examining not just the entire tissue but specific cell types in any tissue where inflammatory cells are abundant or where there are a number of different cell types present. Likewise in disease pathologies including endometriotic lesions, the diseased cells of interest, are surrounded by healthy tissue elements and the cell types of interest may constitute only a small proportion of the volume of the tissue biopsy sample. Laser capture microdissection (LCM) offers a means to overcome inaccuracies arising from analysis of tissues in which there is considerable cellular heterogeneity or in which leukocytes are abundant.

The strengths of two new technologies, LCM and gene array, were utilized in the present study to achieve two main aims. Firstly, to identify chemokines that are differently expressed in glands in eutopic endometrium from normal women and those with endometriosis and secondly, to establish whether these chemokines are also present in glands in endometriotic lesions. Understanding the pathophysiology of chemokine secretion in endometriosis is important as it may offer a novel area of therapeutic intervention. Inhibiting the secretion or action of specific chemokines or blocking their receptors could prevent the inappropriate recruitment of leukocytes to sites of endometriosis-related inflammation. This approach could be considered to act upstream of anti-inflammatory therapies available today which, for the most part, act on the cells already at the site of inflammation.

## Methods

### Patient details and tissue collection

Patients undergoing laparoscopy for pelvic pain, investigation of infertility or tubal ligation were recruited at Monash Surgical Private Hospital and at Monash Medical Centre, Moorabbin and Cranbourne Campus, all in greater Melbourne, Australia. Ethical approval was obtained from appropriate institutional ethics committees and informed consent was obtained prior to surgery.

Control subjects were those with no laparoscopic evidence of endometriosis. Endometriosis patients were assessed according to the revised American Society of Reproductive Medicine classification system [29]. Eutopic endometrium was obtained from both control and endometriosis patients by standard curettage. Ectopic endometriotic tissue was obtained via laparoscopic excision. Areas biopsied included peritoneum, uterosacral ligaments, and the ovary (endometriomata). Women on any hormonal preparations were excluded from the study. Patient details are provided in Table 1.

**Table 1:** Clinical characteristics of endometriosis subjects.

***Endometriosis Patients***					
**Case reference**	**Age (yr)**	**AFS stage**	**Sites of endometriotic lesions**	**Gravidity**	**Parity**

1*^#^	40	II	Peritoneal	6	4
2*^#^	28	I	Uterosacral	0	0
3*^#^	28	II	Peritoneal, Uterosacral	0	0
4*	29	II	Peritoneal, Uterosacral	0	0
5	27	V	Uterosacral	0	0
6	37	V	Ovarian	1	1
7	28	V	Ovarian, Uterosacral, Pouch of Douglas	1	0
8	35	III	Ovarian	1	1
9	37	IV	Peritoneal	0	0
10	37	II	Peritoneal	7	4

***Control Subjects***					

**Case reference**	**Age (yr)**	**AFS stage**	**Sites of endometriotic lesions**	**Gravidity**	**Parity**

1*^#^	42	0	-	0	0
2*^#^	46	0	-	3	3
3*^#^	30	0	-	4	2
4*	32	0	-	0	0

AFS, American Fertility Society

*used in cDNA array analysis (amplified RNA)

^# ^used in real-time PCR analysis (unamplified RNA)

Eutopic endometrial tissue sections were designated to menstrual cycle stage by an experienced gynecological pathologist using the established Noyes endometrial dating criteria [30]. For this study, selected tissue samples, derived only from women in the mid-secretory phase (POD 6–10) were assessed for chemokine expression. Endometrial curettings (eutopic tissue) and endometriotic biopsies were snap frozen immediately at -80°C in OCT embedding medium (Sakura Finetek USA Inc., Torrance CA, USA) and stored at -80°C until use.

### Tissue preparation for laser capture microdissection

Sectioning of frozen tissue samples and processing of sections were performed using methods described previously [31]. Frozen tissue blocks were maintained at -20°C and sectioned at 10 μm, and sections adhered to sterile glass slides (uncharged and uncoated; Objektträger, HD Scientific, Melbourne, Australia) by gentle warming of the slide undersurface and then refreezing at -20°C. Sections were then fixed in cold (0°C) acetone for 1 min, stained with HistoGene (Arcturus Bioscience Inc., CA, USA) for 1 min and dehydrated in 95% ethanol (1 wash, 30 sec), 100% ethanol (three washes, 30 sec each) and xylene (two washes, 5 min each). Sections were then allowed to air dry at room temperature and used for laser capture microdissection.

### Laser capture microdissection

Laser capture microdissection (LCM) was used for the isolation of epithelial glands from endometrial eutopic tissue samples. Frozen tissue sections from patients with endometriosis (n = 4, marked * in Table 1) and control subjects (n = 4) were used for LCM. The PALM^® ^MicroLaser Microdissection System (P.A.L.M. MicroLaser Technologies AG, Burnried, Germany) was utilized. Staining of frozen sections with HistoGene™ (Arcturus Bioscience Inc., CA, USA) enabled the precise selection of the glandular structures. The laser dissected glands were then ejected off the slide with a single defocused laser pulse and catapulted directly into the cap of a microfuge tube containing 10 μl droplet of TRIzol (Gibco BRL, Rockville, USA). For each patient, epithelial glands were laser-captured from approximately 20 tissue sections

### RNA extraction and purification

Total RNA was isolated from each LCM sample using the TRIzol method according to manufacturer's instructions (Gibco BRL, Rockville, USA) following an initial incubation with glycogen (250 ng/μl) and TRIzol reagent for 30 min at room temperature to improve yields. RNA samples were stored at -80°C.

### RNA quantification

The RNA concentration for each sample was determined with the RiboGreen fluorescence RNA assay (Molecular Probes Europe BV, Leiden, The Netherlands) with an *Escherichia coli *ribosomal RNA preparation of known concentration as standard and RiboGreen reagent at a final dilution of 1:500. A standard curve of 0–80 ng/well in a 96-well flat-bottomed plate was employed. This assay gave an intra-assay coefficient of variance (CV), as defined by the repeated analysis of a single RNA sample, of 3.3% (n = 16) and an inter-assay variation of 10.3% (n = 14). To assess RNA quality and tissue specificity, RNA samples were reverse transcribed and primers specific for a mid-secretory phase endometrial gland-specific gene (HtrA3) used for PCR amplification as previously described [32].

### RNA amplification

Amplification of mRNA was performed to achieve the required amount of RNA for cDNA gene arrays. To obtain a representative molecular profile comparison of eutopic endometrium in each of control and endometriosis subject groups, 5 ng of LCM glandular epithelial cell-derived RNA from each of the 4 subjects in the group were pooled. T7 specific RNA amplification of mRNA was performed with the Arcturus HS RNA Amplification Kit (Arcturus) according to manufacturer's instructions. Final RNA yields following amplification, ranged from 0.5 μg to 1.5μg.

### Gene array

Gene profiling was conducted using the GeArray Q Series Human Chemokine and Receptor Gene Array (SuperArray Bioscience Corp., Bethesda, USA) consisting of 77 chemokine and chemokine receptor cDNA probes, printed in quadruplicate on a nylon membrane. Glyceraldehyde-3-phosphate dehydrogenase (GAPDH), cyclophilin and β-actin were included as positive controls and PUC18 plasmid DNA as a negative control. The gene array experiments were performed as previously described [27].

For each array experiment, 100 ng amplified RNA was reverse transcribed to cDNA using Moloney murine leukemia virus-reverse transcriptase (MLTV-RTase) (Promega, Annandale, NSW, Australia) in the presence of biotinylated uridine 5-triphosphate (udTP) (Roche), and incubated overnight in hybridization buffer (SuperArray) containing 100 μg/ml denatured sheared salmon sperm DNA (Invitrogen Australia, Pty. Ltd., Mount Waverley, Vic, Australia) with pre-hybridized nylon arrays at 68°C. Post-hybridization washes with saline/sodium citrate/sodium dodecyl sulfate (SSC/SDS) were also conducted at 68°C. Positive cDNA binding was detected by application of a streptavidin-alkaline phosphatase conjugate in blocking solution and chemiluminescent substrate, CDP-Star as supplied with the arrays (SuperArray).

Membranes were exposed to X-ray film for a range of times between 2 sec and 5 min, to ensure quantitation during the linear phase of the reaction. Arrays for the two subject groups were conducted simultaneously, and the entire experiment was then repeated. X-ray films were scanned at high resolution and densitometrically analyzed using GelDoc software (Bio-Rad Laboratories, Regents Park, NSW, Australia). Chemokine signals were normalized for background signal intensity, to correct for exposure times. Gene expression for each array was also normalised to cyclophilin expression levels. Fold changes in gene expression of 2-fold or higher were considered as significantly different.

### Real time RT-PCR

To confirm array data and to determine the variability between subjects, real time RT-PCR was performed on unamplified RNA derived from individual LCM samples from endometriosis and control groups (n = 3 per group: insufficient starting material was available for the remaining 2 samples). Total unamplified RNA (5 ng) was reverse transcribed using avian myeloblastosis virus-reverse transcriptase (AMV-RTase) (Promega, NSW, Australia) and 1 ng random hexanucleotide primers (Amersham Biosciences, NJ, USA), and the cDNA generated was subsequently amplified by PCR for specific primer pairs encoding CCL16, CCL21 and 18S RNA genes (for primer sequence refer to [27]).

The Roche Light Cycler Real-time PCR system was used (Roche Diagnostics Australia Pty Ltd, NSW, Australia) where 4 μl cDNA (diluted 1 in 10) was added to a master mix including SYBR Green I, deoxynucleotide triphosphates, Taq polymerase enzyme, optimized concentrations of MgCl_2_, and specific primers (0.5 pmol/μl; Sigma Genosys, Australia Pty. Ltd., NSW, Australia). An initial denaturing step was performed for 10 min at 95°C, before 40 cycles of 95°C for 15 sec, 55–66°C for 5 sec (annealing temperature specific to primer pair [27]) and 72°C for 10 sec. All samples to be compared were included within the same run and the entire PCR experiment was performed in duplicate.

Expression of mRNA was quantitated by comparison with a 6-point standard curve of serially diluted (10-fold) cDNA standards specific to the gene product. The standard doses ranged between 2.5 ng/μl and 0.5 fg/μl. Fluorescence from incorporation of SYBR green into double-stranded PCR products was monitored continuously during cycling at the end of each elongation phase, and quantitation of mRNA expression was performed when amplified products were in the log-linear phase and parallel to the standards. A quality control (a single endometrial cDNA sample) was included in every run. At the end of each program, melting curve analysis was carried out to ensure specificity of the reaction products. The sizes of the products were confirmed by gel electrophoresis and DNA sequenced to confirm identity for selected samples. Data was normalized for the expression of 18S RNA.

### Immunohistochemistry

For the further verification of gene array and real time RT-PCR data, frozen sections from 3 of the 4 same endometriosis patients and control subjects, along with tissue sections from an additional 8 normal and 7 endometriosis subjects, were used for the immunohistochemical comparison of chemokine (CCL16 and CCL21) protein expression (n = 12 and 10 respectively per group). Glands were not found in the frozen endometriotic lesion of one endometriosis patient who had been included in the array study and this subject was thus excluded from this part of the study. Affinity-purified polyclonal antibodies raised against human CCL21 and CCL16 peptides (Santa Cruz Biotechnology, Santa Cruz, CA, USA) were utilized for immunohistochemistry as previously described [27]. Positive controls (normal eutopic endometrium) previously shown to have positive staining for CCL16 and CCL21, were included in every run. Negative controls were sections of each tissue included on the same slide but with non-immune IgG at the same concentration substituted for the primary antibodies. For immunostaining, 10 μm sections were post-fixed in 10% buffered formalin for 10 min, rehydrated and exposed to microwave antigen retrieval for 5 min before cooling. Primary antibodies were applied overnight (17 ± 1 h) at 4°C, diluted to 4 μg/ml in non-immune block containing 10% horse serum (Sigma-Aldrich, Sydney, Australia) and 2% human serum (in-house) in TBS with 0.1% Tween 20.

Detection of positive binding was performed by the sequential application of biotinylated horse anti-goat IgG (1:200 in nonimmune block; Vector Laboratories, Burlingame, CA, USA) and avidin-biotin-peroxidase conjugate (Dako, Glostrup, Denmark), followed by the substrate diaminobenzidine (Dako) for between 2 and 10 min. Sections were counterstained with Harris' hematoxylin (Sigma), dehydrated and mounted from Histosol with DPX mounting medium (BDH Laboratory Supplies, Poole, UK). Immunostaining was assessed semi-quantitatively by two independent assessors, blind to the identity of subject groups. Blinding was not possible in the assessment of ectopic tissue potentially biasing the results. Staining intensity and heterogeneity for each endometrial compartment (epithelium, stroma, leukocytes and vasculature) was assessed and allocated a score between 0 and 4 where 0 = no stain, 1 = faint staining, 2 = moderate staining, 3 = strong staining and 4 = maximally intense staining.

### Statistical analysis

None of the data were normally distributed and therefore non-parametric tests were used. The direct comparison of two samples from different patients was conducted with the Mann-Whitney U-test. The direct comparison of two samples (eutopic and ectopic endometrium) from the same patient was conducted with the Wilcoxon Matched-Pairs Signed-Ranks Test. For the relationship between two independent interval variables (staining intensities for CCL16 and CCL21) the rank correlation coefficient was used. The significance level was set at a *P *value of < 0.05.

## Results

### Laser capture microdissection

**Figure 1:**
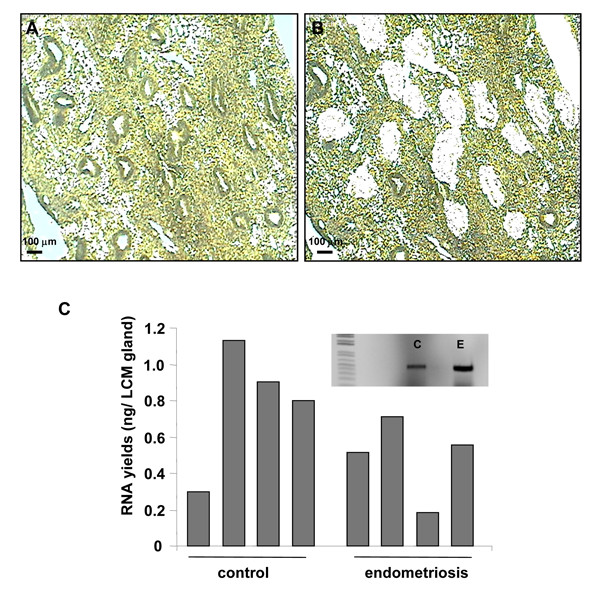
**Laser capture microdissection**. (A) Frozen section of eutopic endometrial tissue from an endometriosis patient (mid-secretory phase) stained with HistoGene™. The epithelial glands were isolated with laser capture microdissection (LCM). (B) Tissue section following LCM demonstrating the precision in dissection and cell capture. (C) RNA yields from microdissected glands expressed as total RNA (ng) per number of gland from each tissue sample. Microdissected glandular RNA samples from control (C, n = 4) and endometriosis (E, n = 4) tissues were separately, reverse transcribed and a uterine epithelial cell-specific gene (HtrA3) was amplified using PCR to assess RNA quality (insert).

Laser capture technology enabled the isolation of glandular epithelial cells from mid-secretory phase endometrial tissue of control and endometriosis subjects (n = 4 per group) (Figure 1A and 1B). For each subject, 150 – 600 glands were microdissected from 10 to 20 individual frozen tissue sections of 10 μm in thickness. Extraction of RNA of the LCM cells produced RNA yields of varying quantities ranging from 0.18 ng to 1.15 ng per glandular structure isolated (Figure 1C). The positive amplification of the epithelial-specific gene product, Htra3, ascertained that glandular epithelial cells had been specifically isolated and RNA integrity retained (Figure 1C).

### Gene array

To provide a global chemokine expression profile in eutopic endometrium from normal women and endometriosis patients, and to overcome patient to patient variability, total RNA extracted from LCM glandular epithelial cells was pooled from 4 individuals in each group (Table 1). Of 77 genes profiled on the cDNA gene arrays, expression of 22 chemokine and receptors were reproducibly (two separate runs) upregulated in pooled endometrium from women with endometriosis compared with controls (Table 2). Downregulation of expression was observed in 11 genes; however only 2 of these demonstrated a 2-fold or higher difference when compared to expression levels in normal endometrium (Table 3). For many upregulated genes, fold increase was not quantifiable, due to the lack of detectable expression in the control pool compared to very high expression in the endometriosis pool. In contrast, the fold-change quantified in genes which were downregulated was relatively small, with the largest difference being a 2.5-fold decrease in CCL28 expression.

**Table 2:** Upregulated genes in glandular epithelial cells of eutopic endometrium: endometriosis compared with controls.

**Regulated gene for chemokines**	**Common name**	**Fold change**	**Associa-ted with receptors**	**Functional expression on immune cells**	**Gene accession**
CCL15	HCC2	++	CCR1, CCR3	T-lymphocytes, monocytes	NM_004167
CCL16	HCC4	++	CCR1	Monocytes	NM_004590
CCL18	PARC	++	unknown	T-lymphocytes	NM_002988
CCL19	MIP-3β	++	CCR7	T-cell and B-lymphocytes.	NM_006274
CCL21	6Ckine	++	CCR7	Leukocytes, endothelial cells	NM_002989
CCL22	MDC	2.2 (1.6, 2.9)	CCR4	Leukocytes, natural killer cells	NM_002990
CCL23	MPIF-1	++	CCR1	T-cell lymphocytes, Monocytes, Neutrophils	NM_001295
CXCL5	ENA-78	2.1 (1.5, 2.6)	CXCR2	Works in conjunction with IL8 to activate neutrophils	NM_002994
CXCL8	IL8	2.2 (1.5, 2.5)	CXCR2	T-cell lymphocyte and neutrophil chemoattractant	NM_000584
CXCL10	I-P10	++	CXCR3	Monocytes, natural killer cells, T-cell lymphocytes	NM_001565
CXCL13	BCA-1	3.0 (1.9, 3.7)	CXCR5	B- and T-cell lymphocytes	NM_006419
XCL1	lymphotactin	++	XCR1	B- and T-cell lymphocytes	NM_005283
XCL2	SCM-1β	++	XCR1	T-cell lymphocytes	NM_003175
CX_3_CL1	Fractalkine	16.4 (2.2, 30.6)	CX_3_CR1	T cells, monocytes	NM_002996

**Receptors**			**Ligand**		

CCR1/CD234	Duffy antigen receptor for chemokines (DARC)	2.2 (1.5, 2.6)	CXCL1, CXCL5, CXCL8	Erythrocytes, leukocytes, Endothelial cells	NM_002036
CCR10	GPR2	++	CCL27	T-cells	NM_016602
CXCR2	IL-8Rβ	++	CXCL8	Neutrophils	NM_001557
CXCR4	fusin	++	CXCL12	T-and B-cell lymphocytes, monocytes, macrophages	NM_003467
CXCR5	BLR1	2.5 (2, 2.8)	CXCL13	B- and T-cell lymphocytes	NM_001716
CXCR6	TYMSTR	5.8 (4.1, 7.5)	CXCL16	T-cell lymphocytes	NM_006564
XCR1	GPR55	++	XCL1 XCL2	T cells, mast cells, monocytes, macrophages	NM_005283
LTB4R*	GPR16	1.9 (1.4, 42.2)	leukotriene B4 (LTB4)	Neutrophils	NM_181657

++ Fold upregulation > 20 fold; in most cases cDNA hybridization signal was undetectable in control arrays but clearly detectable in endometriosis arrays. Where difference in expression was quantifiable and variable between the 2 arrays, fold change is presented in *parentheses*.

*not a chemokine receptor, is a potent chemoattractant and survival factor for neutrophilic polymorphonuclear (PMN) leukocytes (59, 60)

**Table 3:** Downregulated genes in glandular epithelial cells of eutopic endometrium: endometriosis compared with controls.

**Regulated gene for chemokines**	**Common name**	**Fold change**	**Associated receptor**	**Functional expression on immune cells**	**Gene accession**
CCL28	MEC	2.5 (1.1 – 1.7)	CCR10	T-cell lymphocytes, Eosinophils,	NM_019846
CCL4	MIP1β/ACT-2	2.0 (1.3 – 3.3)	CCR6	B- and T-cell lymphocytes, neutrophils, macrophages, monocytes	NM_002984

### Real-time RT PCR

**Figure 2:**
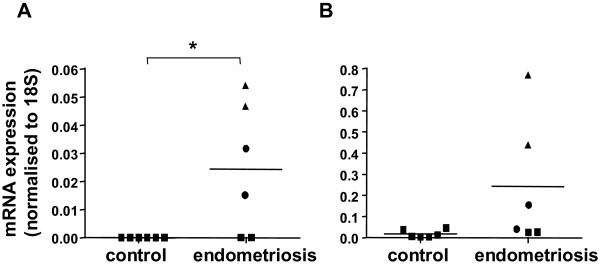
**Real-time PCR quantitation of CCL16 and CCL21 mRNA expression in control and endometriosis subjects**. (A) mRNA expression of CCL16 and (B) CCL21 was measured in glandular RNA samples from individuals in control (■) and endometriosis cohorts (■, ●, ▲ representing the 3 patients' individual data). Data is presented as relative units where chemokine expression has been normalized to 18S RNA expression for each sample (n = 3 per group, RT-PCR performed in duplicates). The average value for each subject group is indicated with a horizontal line. * denotes a statistically significant difference between the 2 groups (*P *= 0.049, Mann-Whitney U-test).

To verify the results obtained from cDNA array experiments and to establish the variability between individuals, two candidate genes (CCL16 and CCL21, also known as HCC-4 and 6C-kine respectively) that had not previously been identified as associated with endometriosis, were selected for further analysis using quantitative real-time RT-PCR on individual unamplified RNA samples of glands from eutopic tissue (patients and controls: n = 3 subjects per cohort). All 3 samples used for each cohort, in this analysis, were included in cDNA array analysis. Selection of the candidate genes was based on the marked upregulation of mRNA levels on the cDNA arrays. These chemokines have been previously characterised in normal cycling human endometrium in our laboratory [27], the mRNA for CCL16 and CCL21 is maximal in entire endometrium during the mid-secretory phase of the menstrual cycle. In this study, mRNA expression for CCL16 was below the detection limit in the LCM glands from all 3 controls and 1 of the 3 endometriosis patient samples (Figure 2A). Where detectable, expression levels ranged from 0.01 – 0.05 fg/pg 18S RNA. Overall there was a significant elevation in eutopic endometrial glandular CCL16 expression in women with endometriosis compared to controls (n = 3; *P *= 0.049).

CCL21 mRNA was more abundant than that of CCL16 and was markedly elevated in eutopic glands from one endometriosis patient (mean, 0.61 fg/pg 18S RNA) (Figure 2B). The levels of CCL21 observed in the remaining endometriosis patients ranged from 0.02 – 0.19 fg/pg 18S RNA. In the control cohort, CCL21 mRNA expression ranged between 0.003 – 0.045 fg/pg 18S RNA. These combined data clearly show that the expression of CCL16 and CCL21 is disturbed at least in some patients with endometriosis. In women without endometriosis the expression was consistently low.

### Chemokine immunohistochemistry in control and endometriosis eutopic tissue

**Figure 3:**
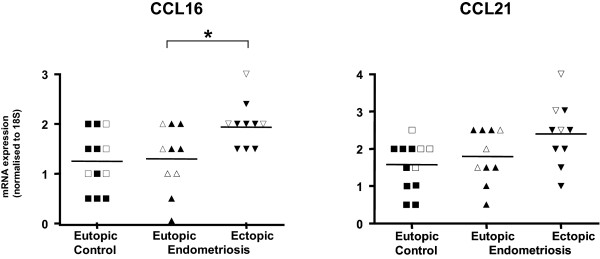
**Immunostaining for CCL16 and CCL21 in eutopic and ectopic tissue from control and endometriosis subjects**. Staining intensity for (A) CCL16 and (B) CCL21 in glandular epithelium of normal mid secretory endometrium (n = 12) (■) and in glandular epithelium in paired eutopic (▲) and ectopic (▼) endometrium from women with endometriosis (n = 10), also in the mid-secretory phase of the menstrual cycle. Open symbols represent subjects whose samples were used in the gene array study. Staining intensity was scored on a scale of 0 (no stain) to 4 (intense stain). Scores are shown for individual samples. Bars represent mean values. * denotes a statistical difference between 2 samples from the same patients (*P *= 0.047, Wilcoxon Matched-Pairs Signed-Ranks Test).

**Figure 4:**
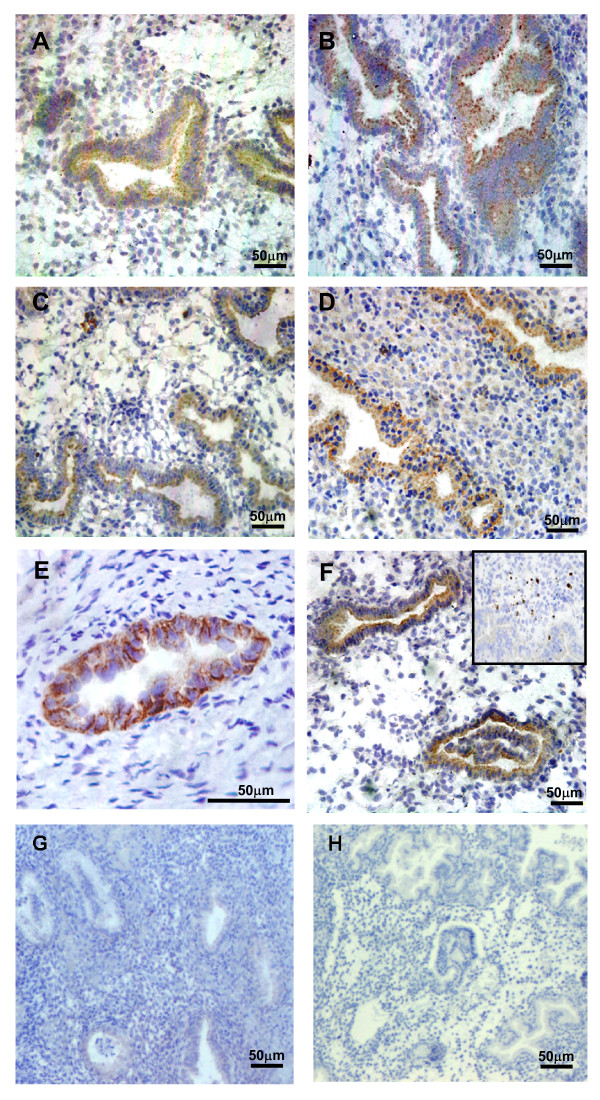
**Immunostaining for CCL16 and CCL21**. Representative tissues immunostained for CCL16 (A, C, E) and CCL21 (B, D, F). CCL16 and CCL21 staining, in control eutopic endometrial tissue (A, B), eutopic tissue (C, D), and matched ectopic lesions (E, F) from the same endometriosis patient. Leukocytes stained for CCL21 are shown in the insert in (F). (G, H) Endometrial sections with non-immune IgG substituted for the primary antibodies, as negative controls. Scale bars represent 200 μm.

Since the findings from the cDNA array and real time RT-PCR experiments supported a role for CCL21 and CCL16 in at least some women with endometriosis, immunohistochemistry was performed to establish the presence and cellular location of the protein for these 2 chemokines in eutopic and ectopic endometrial samples. Immunoreactivity for both chemokines was evident in glandular epithelial cells but absent from stromal cells and was overall in accord with the real-time PCR quantitation. CCL16 protein was detected in the glandular epithelium of all but one of the 32 tissues examined and CCL21 protein was evident in glands in all tissues. However, staining was variable between individual tissues even within groups (Figure 3). No significant differences were detected for either CCL16 or CCL21 between mean glandular staining intensity in endometrium from normal women compared with eutopic endometrium from women with endometriosis (Figure 3). The staining pattern in some glands was predominantly apical (Figures 4A–C) suggesting secretion of the chemokine into the uterine lumen. Within each tissue, all glands were equally stained and staining was present in all cells in each gland (Figures 4A–D).

### Chemokine immunohistochemistry in matched eutopic and ectopic endometriosis tissue

Comparisons were also made between the immunostaining in matched eutopic and ectopic endometrium in endometriosis patients. Positive staining was present in ectopic tissue for both CCL16 and CCL21 (Figures 3 and 4). In both cases, chemokines specifically localised in epithelial cells of glandular structures in ectopic lesions derived from ovarian, uterosacral ligaments and peritoneal sites. In some ectopic peritoneal lesions, large irregular cyst-like structures were present and strong staining for both CCL16 and CCL21 was apparent in glandular epithelial cells and in secretions within the cyst. In other peritoneal lesions, but more commonly, in ovarian lesions, glandular formation was very structured, closely resembling eutopic tissue morphology. Very strong staining was seen in glands in some ectopic lesions (one lesion for CCL16 (Figure 4E) and three for CCL21). Overall glandular staining intensity was higher in ectopic tissue than in eutopic tissue and this reached significance (*P *= 0.047) for CCL16. Importantly, there was a high degree of correlation between staining for CCL16 and CCL21 in each sample (Rank correlation coefficient R = 0.81, *P *< 0.001).

Leukocytes were evident in most tissues and stained strongly for CCL16 and CCL21: these were highly abundant in some lesions (Figure 4F insert) emphasizing the importance of laser capture microdissection in this study. Inclusion of leukocytes in the original cDNA array analysis would have skewed the data. As negative controls, endometrial sections with non-immune IgG substituted for the primary antibodies, demonstrated no localised staining (Figures 4G–H).

## Discussion

This study used laser capture dissection and pathway-specific gene array analysis to determine chemokines that may be expressed at elevated levels during the mid-secretory phase in the glandular epithelium of the endometrium of women with endometriosis when compared to controls. Using pooled samples, 22 genes were markedly upregulated while only 2 genes were slightly downregulated in the endometriosis cohort. Real-time PCR verified that there were differences in the mRNA encoding CCL16 and CCL21, the two chemokines selected for further study, but that there was considerable heterogeneity in expression in individual endometriosis subjects. This heterogeneity was confirmed at the protein level. Furthermore, immunoreactive CCL16 was more intensely stained in epithelium in ectopic endometriosis lesions compared with matched eutopic tissue, and there was a similar trend for CCL21. Indeed, there was high correlation between staining for these two chemokines in each sample. This data thus adds CCL16 and CCL21 to the chemokines found to be elevated in endometriosis supporting a role for chemokines in the pathogenesis of endometriosis. Given the small patient cohort assessed, definitive conclusions cannot be drawn: however the current study provides preliminary data on the involvement of previously unidentified chemokines in the pathogenesis of endometriosis.

Despite the technical difficulties in obtaining sufficient quantities of mRNA with LCM, the current study clearly highlights the importance of laser capture microdissection for the accurate analysis of cell-specific gene expression in heterogeneous tissues such as endometrium. In this tissue, epithelial cells are the predominant source of chemokines [27], many of which are maximally expressed in the mid secretory phase. However, both decidualized stromal cells (found only in late secretory endometrium) and leukocytes are variably abundant in endometrium throughout the menstrual cycle and are additional sources of chemokines. By isolation and initial analysis of glandular epithelium from endometrium, we have been able to identify epithelial-derived chemokines and have excluded the variability and likely spurious results that would have been caused by the presence of infiltrating leukocytes, which also produce chemokines [27].

Laser capture microdissection and RNA amplification technology have been used previously for gene expression profiling of the glandular epithelium in endometriosis patients and aberrant expression was noted in genes relating to oxidative stress and focal adhesion and in genes linked to Wnt, PI3K and RAS/RAF/MAPK signalling [33-35]. As there are no reports of chemokine expression profiling in endometriosis, the present study is the first to provide a comprehensive list of potential candidate chemokines and receptors in laser-captured glandular epithelium in eutopic endometrium from women with endometriosis.

Selected chemokines identified as being of potential interest from the array analysis were further examined by semi-quantitative real-time PCR analysis. Importantly, individual samples were assessed at this stage, to establish not only the variability between the two study groups, but also that between individuals. In laser captured glands from control subjects, CCL16 and CCL21 mRNA were uniformly undetectable or at low levels. In contrast, in women with endometriosis, eutopic CCL16 and CCL21 mRNA levels were increased in some subjects but with significant patient-to-patient variability. This variability could be attributed both to the heterogeneity of the disease, both in cause and phenotype and also to differences in endometrial morphology from individual to individual. Subsequent immunohistochemical examination of the protein however, showed somewhat different results. More tissues were used for this analysis and staining intensities of the two chemokines in the glands were very variable between individuals. Interestingly the major differences in protein were seen between eutopic and ectopic tissues in the same women, with overall higher staining intensity in ectopic tissue. The discrepancy between mRNA and protein levels may reflect either rapid clearance of mRNA or the stability of immunoreactive protein.

The precise roles of CCL16 and CCL21 in the endometrium are currently unknown, but there is considerable information regarding their functions in immunological disease and cancer. CCL16 is specifically chemotactic for monocytes [36-38]. It increases antigen presentation of macrophages, enhances T-cell cytotoxicity and stimulates their production of a number of inflammatory-type cytokines (IL-1β, TNFα, IL-12) [37]. A recent study showed that CCL16 stimulated cell migration of human osteogenic sarcoma cells expressing CCR1 [39]. It is also found to be overexpressed in synovial tissue from patients with inflammatory joint disease [40]. CCL16 has low sequence homology (30%) to other CCR1-dependent chemokines, and significant structural differences, particularly in the N-terminal region required for receptor activation [41]. This may explain why CCL16, although binding to a non-specific receptor, triggers a unique signalling cascade and provides evidence of differential regulatory mechanisms employed by chemokines to illicit cellular responses.

CCL21 has known functions in homeostasis, in the reconstitution of lymphocytes and in immuno-surveillance [42]. It activates T-cells and lymphocytes [43], attracts both T- and B-cells and dendritic cells (DC) to lymphoid tissues through its receptor CCR7. It is essential for the priming of naive T-cells in the initiation phase of the immune response [44,45]. CCL21 has also been implicated in the abnormal adhesion and migration of CD34^+ ^cells in leukemia [46]. How an elevation of CCL21 may contribute to endometriosis is not certain. However, elevations in expression of this normally constitutive chemokine have been reported in cases of chronic inflammation/pathology and autoimmune diseases where a role in inappropriate recruitment of naïve T-cells to non-lymphoid tissue has been proposed [47,48]. Interestingly, endometriosis has been likened to an autoimmune disease [49].

In addition to their upregulation in eutopic endometrium from women with endometriosis, examination of CCL16 and CCL21 in ectopic tissues demonstrated a significant increase in protein relative to expression in eutopic tissue derived from the same individual. A similar observation has been documented recently with the induction of endometriosis in a baboon model with no prior disease [50]. Following the inoculation of endometriosis tissue into the peritoneal cavity of the animal, a concomitant increase in cysteine-rich angiogenic inducer 61 (*CYR61*) gene expression was measured both in eutopic and ectopic tissues. This observation suggests it is the presence of the endometriotic lesions, which directly influences the local environment in the endometrium. In our immunolocalization study of the ectopic lesions, typical large cyst-like structures containing much cellular debris/secretion strongly stained for the chemokines, suggesting secretion of chemokines apically from the epithelial cells. However, the level of chemokine expression did not correlate with the severity of the endometriosis.

CXCL8 (IL-8), another chemokine whose mRNA was increased in endometrial glands from women with endometriosis in our array study, has previously been studied in depth with regard to a role in endometriosis [51], further substantiating our gene array results. CXCL-8 levels are elevated in peritoneal fluid in women with endometriosis [51-53]. Furthermore, glandular epithelial cell localisation and upregulation of immunoreactive CXCR1 and CXCR2, which bind CXCL8, has been demonstrated in eutopic and ectopic tissue sections from endometriosis patients when compared to controls [54].

The data from this pathway-specific gene array thus provides new evidence for the broad dysregulation of chemokine expression in both eutopic endometrial epithelium and ectopic lesions of women with endometriosis. The glandular chemokines which were upregulated are known to recruit and activate natural killer cells, T and B lymphocytes, monocytes and neutrophils. Collectively, such upregulation of chemokines and receptors, in endometriosis patients may demonstrate an increased ability of the endometrium and endometriotic lesions to recruit leukocytes and thus exacerbate the inflammatory response.

Aberrant chemokine expression in the uterine lumen may interfere with blastocyst implantation and hence provide one rationale for the infertility that is often associated with endometriosis. The invasive trophoblast and the eutopic endometrium strongly express chemokine receptors (CCR1, CCR10, CCR5, CCR7, CXCR4, CXCR6 and XCR) and chemokines contribute to the positioning, adhesion and migration of trophoblast [55-58]. Chemokines also have non-immune functions during tissue remodelling and in many diseases through upregulation of adhesion molecule expression, angiogenesis, cell proliferation and motility [59]. The higher abundance of chemokines in endometriosis may facilitate tissue adherence of retrograde menstrual tissue, increase epithelial cell proliferation and remodelling to form glandular structures, and stimulate tissue breakdown and bleeding. There is some functional evidence in support of a role of chemokines in increasing tissue adherence. In a recent study, treatment of mice with a broad-spectrum chemokine inhibitor (NR58-3.14.3) reduced the size and number of post-operative intra-peritoneal adhesions [60] and in particular, a reduction in CD45+ inflammatory cell accumulation within the adhesions was observed.

## Conclusion

In conclusion, the current study has identified potential new candidate molecules from the chemokine family that may be important in the pathogenesis of endometriosis. The use of laser capture microdissection was fundamental to identify only those chemokines and chemokine receptors derived from the epithelial compartment. Further, two novel candidate molecules, CCL16 and CCL21, not previously linked to endometriosis have been validated. This data supports the hypothesis that the inflammatory phenotype observed in endometriosis may involve the activation of chemokine cascades.

## Competing interests

The author(s) declare that they have no competing interests.

## Authors' contributions

ALC carried out tissue collection, laser capture microdissection molecular studies, analysed all data and drafted the manuscript. ASM participated in the design and coordination of the study, recruited patients, performed surgery including tissue excision, performed laser capture microdissection and helped to draft the manuscript. RLJ participated in the design of the study and helped to draft the manuscript. NJH and JZ performed the immunohistochemistry. LAS participated in the conception of the study, its design and coordination and helped to draft the manuscript. LR participated in the conception of the study, its design and coordination, performed the statistical analysis and helped to draft the manuscript.

All authors read and approved the final manuscript.
